# Examining the influence of problematic internet use on sleep quality in Chinese adolescents: a study using the extended Stressor-Strain-Outcome (SSO) model

**DOI:** 10.3389/fpsyg.2024.1447852

**Published:** 2024-08-14

**Authors:** Zhuliu Gong, Yi Guo, Siyuan Fan, Xinying Sun, Yibo Wu

**Affiliations:** ^1^School of Journalism and Communication, Chongqing University, Chongqing, China; ^2^Department of Preventive Medicine, Yanjing Medical College, Capital Medical University, Beijing, China; ^3^School of Public Health, Peking University, Beijing, China

**Keywords:** sleep quality, problematic internet use, adolescent, China, stress

## Abstract

**Objective:**

This study aims to explore how problematic internet use may affect the sleep quality of Chinese adolescents using the extended Stressor-Strain-Outcome (SSO) model. The model posits that stressors indirectly influence behavioral outcomes through the mediation of emotional and physiological strain responses.

**Method:**

A sample of 4,365 adolescents in China participated in this study, which utilized questionnaires and other methods to develop a novel SSO model. In this model, problematic internet use was considered as the stressor, anxiety as an indicator of tension, and sleep quality as the ultimate outcome. Family health was introduced as a moderating variable.

**Results:**

The study found that problematic internet use can significantly and positively predict adolescents’ anxiety (*β* = 0.132, *p* < 0.001) and sleep quality (*β* = 0.362, *p* < 0.001). Furthermore, anxiety was identified as a significant mediating factor between problematic internet use and sleep quality [Effect = 0.066, *p <* 0.05, 95% CI (0.014, −0.0018)]. Family health was observed to have a moderating effect on the relationship between problematic internet use and anxiety (*β* = −0.075, *p* < 0.001).

**Conclusion:**

The research indicates that problematic internet use not only directly increases individual anxiety as a stressor but also indirectly impacts sleep quality by exacerbating anxiety. However, a positive family health status can effectively moderate the adverse impact of problematic internet use on anxiety.

## 1 Introduction

Sleep quality significantly impacts the physical and mental well-being of adolescents. The invention of electric light in the 19th century marked a pivotal shift in people’s perception of sleep, leading to what can be described as a “sleep war” and disrupting the natural rhythms of daily life (Haig, 2018, p. 87).

According to the *China Sleep Research Report (2022)* released by the Chinese Sleep Research Society, 67.03% of primary and secondary school students now sleep for 8 hours or less, compared to 62.9% in 2019. This data indicates a growing trend of insufficient sleep in adolescents. These sleep issues not only affect adolescents’ attention, judgment, and memory but also have detrimental effects on their mental health and weaken their immune system, potentially leading to various diseases (Hertenstein et al., 2019; Delrosso et al., 2020; Kansagra, 2020; Narmandakh et al., 2020; Gautam et al., 2021). Given the evolving data and persistent problems, two critical questions arise: Has the “sleep war” truly come to an end? If not, what are the real factors impacting the sleep quality of adolescents?

Previous studies have revealed that adolescents’ sleep quality is influenced by a combination of physiological and social factors. Physiological factors such as sleep apnea, melatonin secretion disorders, and obesity can lead to inadequate sleep. Additionally, social factors play a significant role, including psychological anxiety (Willis and Gregory, 2015; Blake et al., 2018; Zhang L. G. et al., 2023; Zhang Y. et al., 2023), neighborhood environment (Mayne et al., 2021), academic pressure (Sharman and Illingworth, 2019), and social networking (Carvalho-Mendes et al., 2020). With the advancement of internet and media technology, sleep issues have become more complex. Many studies have delved into the intricate relationship between media exposure, problematic internet use, and sleep, particularly focusing on the impact of problematic internet use on adolescent poor sleep quality (Hale et al., 2019; Hamilton et al., 2020; Kortesoja et al., 2023).

Problematic internet use, also known as internet addiction or excessive internet use, can have detrimental effects on the psychological well-being, social behavior, and emotional function of the general public (Salmela-Aro et al., 2016; Yeon-Jin et al., 2018). For adolescents, the allure of the internet’s “dark side” can lead to compulsive and repetitive digital activities, resulting in negative consequences such as anxiety, loneliness, depression, and sleep disorders (Bozoglan et al., 2014; Salmela-Aro et al., 2016; Ranjan et al., 2021). Research indicates that problematic internet use is often associated with increased usage time and its dependency, signifying a chronic lack of self-control and an increased likelihood of insufficient sleep (Vernon et al., 2015). Moreover, prolonged online habits (Higuchi et al., 2005) can lead to excessive stress, stimulating the central nervous system and further compromising sleep quality (Öks et al., 2018; Wang et al., 2022). Additionally, problematic internet use has been linked to a higher incidence of sleep disorders, significantly impacting daily functioning (Wolniczak et al., 2013). In summary, the relationship between problematic internet use and the sleep quality in teenagers is complex and warrants further exploration. Problematic internet use directly impacts the sleep quality of adolescents and has been identified as a precipitating factor influencing anxiety levels in adolescent populations (Thomée et al., 2011). Some scholars have explored how anxiety, in turn, further affects adolescents’ sleep quality (Xiao et al., 2023). While existing research primarily focuses on the causal relationship between problematic internet use and loneliness, and their mutual influence, there is a lack of emphasis on potential mediating and moderating variables between the two.

Problematic internet use can be considered as a stress-precipitating factor leading to poor sleep quality. This study aims to analyze the impact of variables such as problematic internet use on the sleep quality in teenagers, based on the Stressor-Strain-Outcome (SSO) model.

The SSO model comprises three components: stressor, strain, and outcome. It elucidates the relationship between external stress, resulting emotional and physiological reactions, and subsequent negative behaviors (Koeske and Koeske, 1993). Recently, the application of the SSO model has expanded from workplace environments to social media contexts, examining the adverse effects of technological advancements on public behavior (Mick and Fournier, 1998; Cao et al., 2018; Dhir et al., 2018). As the SSO model aligns with the focus of the current study, we propose that problematic internet use (stressor) may lead to anxiety (strain) among teenagers, potentially triggering poor sleep quality (outcome) in this demographic.

This study examines the impact of problematic internet use on adolescents’ anxiety and sleep quality using the SSO (Stimulus-Organism-Response) model as a framework. Our objectives are three-folded. First, testing several research hypotheses related to these variables. Second, elucidating the relationships between problematic internet use, anxiety, and sleep quality in adolescents. Third, provide empirical evidence for the applicability of the SSO model in this context.

*H1*: Problematic internet use positively predicts anxiety.

*H2*: Anxiety positively predicts the quality of sleep.

*H3*: Problematic internet use positively predicts the quality of sleep.

*H4*: Anxiety plays a mediating role between problematic internet use and sleep quality.

Based on a review of existing research, it is evident that social factors are often examined through the lens of individual behavior and emotions of teenagers. There is an implicit assumption that teenagers can autonomously make decisions about their lives. In the contemporary Chinese society, family plays a fundamental role in shaping adolescents’ daily lives. The collective mindset views family as a “safe haven,” often described metaphorically as “the warm shore for the drifting ship of life,” emphasizing its crucial importance. The family unit profoundly influences an individual’s beliefs and behaviors, catalyzing significant personal transformations (Otani et al., 2017). The intergenerational transmission of values and emotions within families serves as ultimate powerful force in driving changes in individual beliefs and behaviors. This influence extends to various aspects of an individual’s well-being, including psychological states and sleep quality (Hale et al., 2015; Maratia et al., 2023). Family Systems Theory characterized a family as an interconnected system with emotional interaction patterns between generations. This theory views family members as interconnected elements within this system (Janet et al., 1982). Building on this concept, this study aims to incorporate family health into the research model to explore its potential impact on anxiety.

*H5*: Family health has a moderating effect between problematic internet use and anxiety.

By validating this hypothesis, the study aims to analyze the impact of problematic internet use on teenagers’ sleep quality, based on the SSO theory. Specifically, it seeks to explore the mediating role of anxiety and the moderating role of family health on anxiety. The sleep condition of teenagers, particularly in China, is currently a subject of significant concern (Liang et al., 2021; Chen et al., 2023; Zhang L. G. et al., 2023; Zhang Y. et al., 2023). Therefore, conducting an in-depth study of the factors influencing teenagers’ sleep quality is essential for their healthy development. While cross-sectional studies cannot establish causality, they can provide a theoretical framework for further investigation in longitudinal and randomized intervention studies.

## 2 Method

### 2.1 Survey method

This study employed a cross-sectional, face-to-face survey design targeting adolescents aged 12–17 years across mainland China. The survey covered 23 provinces, 5 autonomous regions, and 4 municipalities, encompassing 148 cities, 202 districts/counties, 390 townships/towns/streets, and 780 communities/villages. Hong Kong, Macau, and Taiwan regions were not covered. The sampling procedure utilized a multi-stage stratified approach. Sample size for each province, autonomous region, and municipality was determined based on population proportions derived from the Seventh National Population Census data. Subsequently, stepwise sampling was conducted at the city, district/county, township/town/street, and community/village levels.

A total of 4,500 questionnaires were distributed. Following rigorous validity analysis, 4,265 questionnaires were deemed valid, yielding an effective response rate of 94%. This high response rate enhances the generalizability of our findings to the broader adolescent population in China.

Two rounds of pilot studies were conducted in mid-June and early July 2022, each involving 100 participants. Quota sampling was employed, with quota attributes matching those of the formal survey. These pilot studies informed refinements to the questionnaire, enhancing its comprehension and cognitive accessibility for the target population.

The formal survey was administered via the Wenjuanxing platform.^1^ Questionnaires were distributed in a one-on-one, face-to-face format, with participants responding via a provided survey link. Each questionnaire was assigned a unique identifier with the participant’s informed consent. For participants unable to complete the questionnaire independently, survey administrators provided explanations and recorded responses according to the participant’s expressed intentions.

To minimize potential biases during data collection, the following measures were implemented: (1) Strict adherence to research design principles and statistical requirements; (2) Systematic participant registration and coding; (3) Daily briefings for survey administrators to reinforce protocol adherence; (4) Weekly evaluations of collected questionnaires by the research team, addressing issues promptly and ensuring compliance across all survey teams.

Post-collection, two independent researchers conducted back-to-back logical checks and data screening to ensure data integrity. This methodological approach, incorporating pilot studies, refined data collection procedures, and rigorous quality control measures, enhances the reliability and validity of the research findings.

This study adhered to strict ethical guidelines to protect participant anonymity and confidentiality. Prior to data collection, participants were provided with comprehensive information regarding the study’s objectives, methodology, data usage, and confidentiality measures. Informed consent was obtained from all participants, who were required to sign confidentiality agreements. To ensure data protection, we implemented robust anonymization and desensitization processes. All interview records and collected data were securely stored with restricted access. Data usage and sharing were strictly controlled in accordance with the confidentiality agreements.

### 2.2 Participants

Adolescents were defined as individuals aged 12–19, based on the study’s criteria. Inclusion criteria included being at least 12 years old, possessing Chinese nationality, being a Chinese permanent resident, voluntarily participating in the study, able to complete the online questionnaire independently or with the help of an investigator, and understanding the questionnaire items. Exclusion criteria involved individuals with impaired consciousness or mental disorders, those participating in other similar research projects, and those unwilling to cooperate.

The study conducted an analysis of the respondents’ basic information using SPSS 22.0 statistical software. The analysis, presented in Table 1, revealed that 48% of the respondents were male, while the proportion of female respondents was slightly higher at 52%. The Han nationality represented the overwhelming majority at 89.2%. In terms of occupation, students accounted for 98.4% of the respondents. Education-wise, bachelor’s/associate’s degree and high school/vocational school were the primary categories, representing 45.6 and 32%, respectively. Additionally, in terms of household registration, 54% of the total respondents were urban residents, while 46% were rural residents, with 2,300 and 1965 individuals in each category, respectively.

**Table 1:** Sociodemographic descriptive statistics table.

Variable	Option	Frequency	Proportion
Gender	Male	2043	48%
	Female	2,222	52%
Ethnic group	Han nationality	3,806	89.2%
	Ethnic minorities	459	10.8%
Current educational stage	None	71	1.6%
	Primary school	122	2.8%
	Junior high school	770	18%
	High school/Technical secondary school	1,366	32%
	College/Bachelor	1936	45.6%
	Master’s degree or above	0	0
Permanent residence	Town	2,300	46%
	County	1965	54%
Age	12	130	3%
	13	191	4.4%
	14	330	7.7%
	15	380	8.9%
	16	494	11.5%
	17	517	12.1%
	18	998	23.3%
	19	1,225	29.1%

### 2.3 Questionnaire design and variable measurement

The process of designing the questionnaire involved several steps. First, a significant amount of relevant literature was collected to determine the research content, understand previous scholars’ questionnaire design, and identify research questions and variables. Second, questions were developed and organized to create the initial questionnaire. Third, a pre-survey test was conducted to assess the questionnaire’s validity and the logic of the questions. Based on the pre-survey results, adjustments were made to finalize the questionnaire for the investigation. The final questionnaire comprised two main parts: the first part focused on demographic variables, including gender, current school stage, household registration, and age, aimed at understanding the general situation of the research participants, especially adolescents. The second part measured various variables (Table 2).

**Table 2:** Validity analysis of various scales.

Validity	KMO	Bartlett’s Test of Sphericity		
		Approximate Chi-Square	df	*p*
Problematic internet use	0.810	9601.21	15	<0.001
Anxiety	0.946	26243.51	21	<0.001
Family health	0.890	30655.10	45	<0.001
Sleep quality	0.702	5717.93	10	<0.001

#### 2.3.1 Problematic internet use scale

In the assessment of problematic internet use in adolescents, the study utilized the Problematic Internet Use Scale developed by Opakunle et al. (2020). This scale comprises three dimensions and six items, which inquire about various aspects of internet use, such as preference for surfing online over sleeping, feelings of tension when unable to access the internet, attempts to reduce online time, concealment of internet usage, complaints from others about excessive online-surfing time, and feelings of anxiety or tension when offline. These questions assess three areas of problematic internet use: obsession, neglect, and control disorder. Responses were scored on a Likert 5-point scale, ranging from never (0 points) to always (4 points), resulting in a total score range of 0–24 points, with a higher score indicating a more severe degree of problematic internet use among adolescents. In this study, the Cronbach’s α coefficient for this scale was 0.820.

#### 2.3.2 Generalized anxiety disorder 7-item scale

In assessing the level of anxiety in adolescents, the study utilized the GAD-7 scale developed by Spitzer et al. (2006). Each item on the scale is scored on a range of 0–3 points, with a higher total score indicating a higher level of anxiety. Specifically, a GAD-7 score of ≥10 serves as the cutoff value for screening. Scores of 5, 10, and 15 represent varying degrees of anxiety, ranging from mild to moderate to severe. In this study, the Cronbach’s α coefficient for this scale was 0.948.

#### 2.3.3 Short-form family health scale

As a reliable measurement tool, the study employed the Short-Form Family Health Scale to assess the family health status of the respondents (Crandall et al., 2020). This scale consists of 10 items covering four dimensions: external social support, healthy lifestyle, health resources, and social/emotional health. Participants used a 5-point Likert scale (1 = strongly disagree and 5 = strongly agree) to indicate their level of agreement with the family-related statements. Following the recoding of reverse-phrased items, reliability testing was conducted, revealing a Cronbach’s α coefficient of 0.827 in this study, indicating highly reliable measurement results.

#### 2.3.4 B-Pittsburgh sleep quality index scale

The study employed the B-PSQI scale to assess the sleep quality of adolescents. This scale, based on the work of Buysse et al. (1989) and further simplified by Sancho-Domingo et al. (2021), focuses on five dimensions: sleep efficiency, subjective sleep quality, sleep duration, sleep disturbances, and sleep latency (Sancho-Domingo et al., 2021). This streamlined version aims to improve measurement efficiency and applicability by reducing the number of items from 18 to 6 and eliminating two dimensions. Participants used a 4-point Likert scoring guideline, with a higher score indicating poorer sleep quality. The reliability analysis of the questionnaire found that the Cronbach’s α coefficient for this scale in the study was 0.784, indicating highly reliable measurement results.

## 3 Results

### 3.1 Validity analysis

Validity analysis aims to assess whether the questions in the questionnaire effectively capture the corresponding indicators or dimensions. It evaluates the reasonableness of the questionnaire’s design and its ability to appropriately reflect the measured variables. Typically, a Kaiser-Meyer-Olkin (KMO) value above 0.7 indicates relatively ideal validity of the scale. Additionally, Bartlett’s test of sphericity is conducted, where a *p*-value less than 0.05 signifies a significant result. The results of the validity analysis are presented in Table 2. The table shows that the KMO values of all scales exceed 0.7, and the *p*-values are all less than 0.05, indicating high validity for all questionnaire scales.

### 3.2 Descriptive statistical analysis

Descriptive statistical analysis is used to investigate the overall distribution of sample data through the analysis of various statistical indicators such as frequency distribution and measures of dispersion. In this study, the key indicators for statistical description were selected based on the actual research objectives, with a focus on frequency and mean. These indicators effectively capture the basic characteristics of the sample in terms of demographic variables and reveal distinct behavioral traits of other variables. This comprehensive analysis provides robust data support for subsequent research.

The detailed results of a descriptive analysis of various respondent variables are presented in Table 3. The analysis indicates that anxiety among adolescents constitutes a significant proportion of 54.2%, with 36.5% experiencing mild anxiety, 11.1% experiencing moderate anxiety, and 4.4% experiencing severe anxiety. These findings highlight the prevalence of anxiety among adolescents, with mild anxiety being the most common type.

**Table 3:** Descriptive analysis of relevant variables.

Degree of anxiety		
Option	Frequency	Proportion
No anxiety	1954	45.8%
Mild anxiety	1,558	36.5%
Moderate anxiety	473	11.1%
Moderate anxiety	190	4.4%

Sleep quality		
Option	Average score	Standard deviation
Time to fall asleep	1.77	0.91
Sleep duration	0.80	0.83
Sleep disorder	1.52	0.83
Sleep efficiency	0.60	0.80
Sleep quality	1.82	0.71

Problematic internet use		
Option	Frequency	Proportion
Obsession	3.25	0.45
Neglect	3.07	0.81
Control disorder	3.44	0.76

The mean total sleep quality score of the sample in this study was 5.78 ± 3.27. According to the Pittsburgh Sleep Quality Index (PSQI) scoring criteria, score exceeding 5 indicate poor sleep quality, with higher scores reflecting more severe sleep disturbances.

Thus, our findings suggest that the average participant in our sample experiences sleep quality issues. Further analysis of specific sleep dimensions revealed concerning trends, particularly regarding sleep duration. A majority of adolescents (57%) reported sleeping less than 7 h per day, which falls below the recommended 8–10 h for this age group (Paruthi et al., 2016).

### 3.3 Correlation analysis

Correlation analysis is used to measure the strength of the relationship between variables, as indicated by the correlation coefficient. This coefficient reflects both the magnitude and direction of the relationship between two variables. Subsequent data analysis relies on the findings of correlation analysis. In this study, the Pearson coefficient was used to assess correlation and to determine the direction of the relationship, whether positive or negative. Details are presented in Table 4.

**Table 4:** Correlation coefficient between variables.

	Problematic internet use	Family health	Anxiety	Sleep quality
Problematic internet use	1			
Family health	0.341**	1		
Anxiety	0.303**	−0.271**	1	
Sleep quality	0.311**	−0.224**	0.342**	1

** Significant correlation at the 0.01 level (two-tailed); * Significant correlation at the 0.05 level (two-tailed).

The data shows a positive correlation between problematic internet use and family health (r = 0.341, *p* < 0.01), as well as a positive correlation between problematic internet use and anxiety (r = 0.303, *p* < 0.01). Additionally, there is a positive correlation between problematic internet use and sleep quality (r = 0.311, *p* < 0.05). Conversely, there is a negative correlation between family health and sleep quality (r = −0.271, *p* < 0.05), and family health also exhibits a negative correlation with anxiety (r = −0.224, *p* < 0.05). It is important to note that there is a positive correlation between anxiety and sleep quality (r = 0.342, *p* < 0.05).

The analysis revealed moderate positive correlations between problematic internet use and family health, anxiety, and sleep quality. Weak to moderate negative correlations were observed between family health and both sleep quality and anxiety levels. Additionally, a moderate positive correlation was found between anxiety and sleep quality. The moderate nature of these correlations provides valuable insights for future research and intervention strategies. It implies that there is potential for modulation and improvement of these relationships through appropriate interventions and methodologies. This finding opens avenues for developing targeted approaches to address problematic internet use, enhance family health, mitigate anxiety, and improve sleep quality among adolescents.

### 3.4 Model validation and results

This study utilized the Partial Least Squares method (PLS) within the structural equation model (SEM) framework to empirically test the research hypotheses. PLS, as a second-generation data analysis technique, offers significant advantages. It does not require data to follow a normal distribution nor imposes strict requirements on sample size. Importantly, PLS is well-suited for exploratory research and testing of new models and theories, allowing for in-depth exploration of emerging phenomena. Given the exploratory nature of research on the impact of problematic internet use on sleep quality, this study believes that the PLS method can better meet the research needs. Therefore, the Smart PLS 4.0 statistical software was employed in this study, and a bootstrapping program with 2,000 samples was run to accurately calculate the path coefficients between constructs. This approach not only enhances the study’s accuracy but also provides robust support for further uncovering the relationship between problematic internet use and sleep quality.

#### 3.4.1 Analysis of structural equation modeling

**Figure 1:**
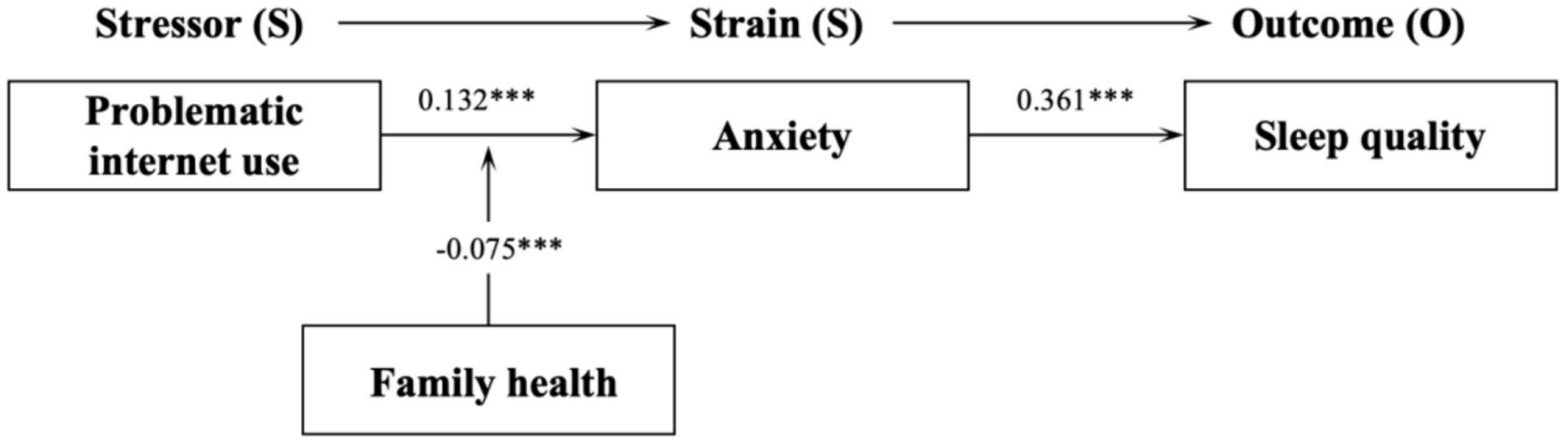
Results of path analysis; * *p* < 0.05; ** *p* < 0.01; *** *p* < 0.001.

Figure 1 presents the results of the Smart PLS 4.0 analysis of the theoretical model. The findings reveal that the structural equation model, including control variables, explains 9.4% of anxiety and 13.1% of sleep quality, indicating strong explanatory power for our theoretical model. Moreover, the path analysis results confirm the direct effects as hypothesized. Specifically, problematic internet use significantly positively predicts anxiety (*β* = 0.132, *p* < 0.001), and anxiety, in turn, is a significant positive predictor of sleep quality (*β* = 0.362, *p* < 0.001). As a result, hypotheses H1, H2, and H3 are all validated. These results imply a close relationship between problematic internet use and adolescents’ level of anxiety, with frequent problematic internet use potentially exacerbating anxiety. This heightened anxiety, in turn, may contribute to a decline in sleep quality.

#### 3.4.2 Testing the mediating effect of anxiety

**Figure 2:**
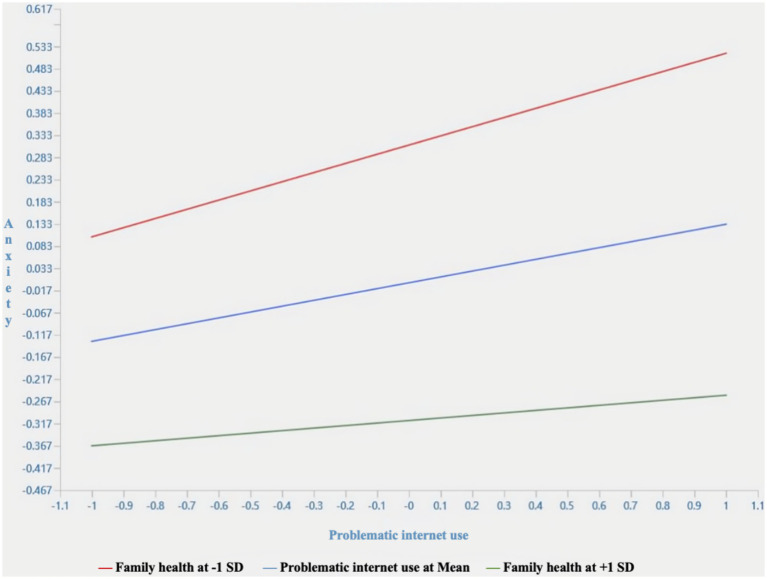
Simple slope chart of family health.

After comparing the Bootstrapping mediation analysis method and the bias-corrected percentile Bootstrapping mediation analysis method (Zhao et al., 2010), this study integrated previous research (Chin et al., 2003) and adopted the Bootstrapping mediation analysis method in SmartPLS 4.0 to conduct the mediation effect test. The test requirement is that 0 should not fall within the 95% confidence interval of the indirect effect, indicating a significant mediating effect; otherwise, the mediating effect is not significant. As can be seen from Table 5, 0 is not within the confidence interval of this bias correction. Therefore, it can be considered that anxiety has a mediating role in the impact of problematic Internet use on adolescents’ sleep quality. Thus, Hypothesis 4 is validated (Figure 2).

**Table 5:** Mediation effect test.

Mediation path	Initial Sample (O)	Standard deviation (STDEV)	T-Statistic (O/STDEV)	Bias-corrected confidence interval		*p*
				2.50%	97.50%	
Problematic internet use -> Anxiety	0.039	0.015	2.539	0.039	0.009	0.011
Anxiety -> Sleep quality	1.692	0.078	21.574	1.692	1.538	0.000
Problematic internet use -> Sleep quality (Direct Effect)	0.146	0.068	2.156	0.146	0.281	0.031
Problematic internet use -> Anxiety -> Sleep quality	0.066	0.026	2.499	0.014	0.018	0.012
Problematic internet use -> Sleep quality (Total Effect)	0.048	0.008	5.619	0.017	0.059	0.000

#### 3.4.3 Test of moderating effect of family health

This study utilized Cohen’s ƒ2 to explore the moderating effect of family health on the relationship between problematic internet use and sleep quality. The ƒ2 value was calculated by comparing the explained variance between models with and without the interaction term. A large moderating effect was indicated when ƒ2 was greater than 0.35, a moderate effect when ƒ2 ranged between 0.35 and 0.15, a small effect when ƒ2 ranged between 0.15 and 0.02, and no moderating effect when ƒ2 was less than 0.02 (Cohen, 1988, p. 22).

In the relationship between problematic internet use and anxiety, with the addition of the interaction term between problematic internet use and family health, the ƒ2 value was 0.098, and the R^2^ increased from 0.094 to 0.131, indicating a certain moderating role of family health. To further investigate the moderating effect of family health on problematic internet use and anxiety, a simple slope graph was plotted between problematic internet use and anxiety based on the mean of family health plus or minus one standard deviation. The results revealed that family health significantly weakened the impact of problematic internet use frequency on anxiety. Specifically, under low family health levels, the positive impact of problematic internet use on anxiety was significantly higher than under high family health levels. As a result, Hypothesis 5 was validated (Figure 2).

## 4 Discussion

Based on the SSO model, this study aims to elucidate the impact of problematic internet use on adolescent sleep quality, and the influence of anxiety and family health on adolescent sleep quality. The research findings indicate that problematic internet use has a detrimental effect on anxiety, leading to a decline in sleep quality. In essence, problematic internet use acts as a stressor, contributing to the generation of negative emotions such as anxiety among adolescents, ultimately resulting in poor sleep quality.

Previous research has predominantly examined the unidirectional impact of anxiety on problematic internet use. Davis’s cognitive-behavioral model of Pathological Internet Use (PIU), for example, proposes anxiety as a contributing factor in the development of PIU (Davis, 2001). Subsequent studies have expanded on Davis’s model, providing evidence that social anxiety, in particular, can precipitate problematic internet use among adolescents and young adults (Chen, 2024; Huan et al., 2014). However, our study differs from these perspectives through rigorous investigation and data analysis. We found that problematic internet use can predict its impact on anxiety. This finding aligns with a limited number of other studies, such as a longitudinal study showing that individuals with problematic internet use are about 2.5 times more likely to experience depressive symptoms compared to those without problematic internet use (Lam and Peng, 2010). Problematic internet use also heightens the risk of anxiety and stress, particularly among adolescents and young people, amplifying their susceptibility to depression and anxiety symptoms. The issues associated with problematic internet use, encompassing obsession, neglect, and control disorders, contribute to heightened anxiety and mental health challenges among adolescents. The stress resulting from these issues exacerbates the erosion of adolescents’ rationality, intensifies self-loss, and obfuscates their genuine needs, ultimately leading to deeper-level anxiety and mental health problems (Moretta and Buodo, 2020; Gioia et al., 2021; Jahan et al., 2021).

We have established a significant positive correlation between anxiety and sleep quality (*β* = 0.362, *p* < 0.001), indicating that higher anxiety levels in adolescents are associated with lower sleep quality, which aligns with previous research (Randler et al., 2016; Lima et al., 2020; Pagano et al., 2023). According to the cognitive model of insomnia, excessive worries, anxiety, fears, and intrusive thoughts can impair sleep quality and worsen insomnia (Harvey, 2002). Tochigi et al. also found that high levels of anxiety and depression in adolescents aged 12 to 18 can delay bedtime, consistent with this model (Tochigi et al., 2016). The state–trait anxiety theory suggests that persistent negative emotions like tension and anxiety can carry over into sleep, contributing to poor sleep quality. This theory also highlights that excessive tension or worry about sleep quality at bedtime can prolong sleep onset and influence sleep quality, corroborating our findings. Our data indicates that students with high anxiety tend to have delayed bedtimes and experience longer sleep onset latency, suggesting that heightened anxiety may lead to poor sleep quality.

Based on these theoretical models and empirical data analysis, we infer that anxiety, as an intrusive thought, is a predisposing factor for insomnia, keeping individuals in a highly aroused state and predisposing them to excessive rumination (You et al., 2021). When individuals fall into depressive emotions, this negative state can lead to difficulties in falling asleep (You et al., 2021). The fear of poor sleep and the struggle to fall asleep when anticipating difficulty falling asleep are common occurrences. At the same time, adolescents with poor sleep quality tend to be overly concerned about their sleep and its consequences, subjectively sensitive to the discrepancy between their sleep status and the significance of tasks, forming negative perceptions of “poor sleep” (Harvey, 2002). Such cognitive biases trigger emotional and physiological changes, ultimately leading to insomnia. In addition, most of the samples selected in this study are students during their academic years, and academic and social anxieties can lead adolescents to pre-sleep rumination (Gong et al., 2024) and have “allergic” reactions that affect their sleep quality.

We have found that problematic internet use significantly and positively predicts the sleep quality of adolescents, with anxiety playing a crucial mediating role in this relationship. The more severe the problematic internet use, the poorer the sleep quality of adolescents. Previous studies have widely recognized that problematic internet use disrupts the balanced operation between the physical body and the natural sleep patterns of adolescents. The short-wavelength light emitted by electronic screens, such as tablets and smartphones, can trick the brain into perceiving it as daytime, inhibiting the production of melatonin and affecting users’ sleep (Chinoy et al., 2018). However, sleep quality and behavior are not solely within the realm of biological research, but also within the purview of psychology, an interdisciplinary field between natural and social sciences. It is increasingly important to establish a cultural normative understanding of sleep from the perspective of media psychology. Research in this area has found that excessive media use before bedtime can misalign users’ perception of time (Chen, 2024), delaying adolescents’ bedtime, triggering cognitive arousal before sleep (Harbard et al., 2016), and potentially causing anxiety related to the “fear of missing out” (Tandon et al., 2022). Combining these factors can potentially form a vicious cycle that impacts adolescents’ sleep quality and overall health (Pagano et al., 2023). Additionally, the sleep disturbance process theory suggests that excessive emotional arousal can significantly impact sleep quality. Some scholars also argue that stress-related hormones, such as cortisol and corticosterone, can disrupt sleep patterns during certain stages of sleep when individuals experience stress (Yan et al., 2010). Our findings further validate the mediating role of anxiety between problematic internet use and adolescents’ sleep quality based on the SSO theory, as indicated by previous studies.

In addition, our research has revealed that family health moderates the impact of problematic internet use on anxiety within the SSO model. Family health encompasses various factors such as external social support, healthy lifestyle, access to health resources, and emotional well-being within the family (Wang et al., 2023). These factors have been identified as protective elements against mental health issues (Cheng et al., 2017). Previous studies have demonstrated that family support can positively influence adolescents’ mental health, offering them a shield during challenging developmental phases (Thomas et al., 2017; Brinksma et al., 2019). Therefore, our inference is that when family members prioritize caring for and supporting each other while maintaining a healthy lifestyle, individuals may reduce their reliance on media, consequently lowering anxiety triggered by problematic internet use. Moreover, high levels of family health may equip family members with better coping mechanisms for anxiety. Effective communication, emotional support, and joint problem-solving within a family environment can help alleviate anxiety. This type of supportive environment contributes to building individuals’ psychological resilience, enabling them to stay composed and level-headed in the face of stress and anxiety. Our study draws from family system theory, particularly the concept of the “triangle,” which illustrates the dynamic interplay between three individuals and its correlation with chronic anxiety (Jakimowicz et al., 2020). Under normal circumstances, a two-person relationship tends to maintain relative stability, with anxiety levels remaining low. However, as anxiety levels escalate, the relationship between the two individuals may experience strain. To address this pressure and anxiety, the involvement of a third party is often necessary, transforming the original two-person relationship into a triangular one. Within the framework of our study, family health serves as this third party, moderating the adverse impact of problematic internet use on adolescent anxiety.

## 5 Limitation

This study not only contributes to the understanding of the relationship between problematic Internet use, anxiety, and sleep quality but also lays a theoretical foundation for potential practical interventions. However, it is important to note that the study has certain limitations. The data analysis in this article is based on a national cross-sectional survey, so caution is advised when inferring causal relationships. Future studies could consider longitudinal designs to monitor changes in problematic Internet use, anxiety, family health, and sleep quality among adolescents over an extended period.

Additionally, there may be inherent issues with subjective assessments in self-reported measurements. Furthermore, as the family is a complex communication system, it is influenced by various demographic variables such as gender, age, education, and income, as well as different family types. Subsequent research efforts could explore the diverse impacts of various family structures on problematic Internet use, anxiety, and sleep quality among adolescents.

## 6 Conclusion

Existing literature primarily focuses on public health, with limited studies from the perspective of media psychology. This study explores the internal mechanism of how problematic internet use affects sleep quality using the Stressor-Strain-Outcome (SSO) model. The findings demonstrate a significant correlation and impact of problematic internet use on anxiety and sleep quality. By developing a theoretical model and conducting empirical research, we establish that problematic internet use notably influences individuals’ anxiety. Importantly, it not only directly impacts sleep quality but also indirectly affects it by triggering anxiety. This discovery reveals the intricate relationship between problematic internet use and sleep quality, offering a fresh angle for further research in related fields.

Furthermore, this study is the first to introduce the significant variable of family health into the SSO model and to examine its role in this relationship. The results indicate that when family health levels are high, the adverse impact of problematic internet use on sleep quality is diminished. This suggests that family support plays a crucial role in alleviating the stress caused by problematic internet use. Therefore, parents should endeavor to create a harmonious and nurturing family environment, actively addressing the psychological and emotional challenges brought about by modernity through effective communication, attention to children’s mental health, and thoughtful management of adolescents’ media exposure. This proactive approach will promote the physical and mental well-being of young people, improve their quality of life and sleep, and establish a strong foundation for their healthy development.

The findings of this study demonstrate a degree of universality and generalizability. The observed associations among problematic internet use, anxiety, and sleep quality suggest broader societal implications, offering valuable insights for further exploration in related fields such as psychology, education, and sociology. Family health emerged as a crucial factor in mitigating the negative effects of problematic internet use. The role of family health may transcend diverse cultural backgrounds and age groups, warranting further investigation. Future research could explore how family health moderates the relationship between problematic internet use and sleep quality across various cultural and socioeconomic contexts, as well as different age cohorts. The intervention strategies proposed in this study, including fostering a harmonious family atmosphere, promoting effective communication, and prioritizing children’s mental health, have potential for broad applicability. These strategies may be relevant not only to our study population but also to other families and educational institutions across diverse settings. Implementation of these strategies could help individuals better cope with the challenges posed by problematic internet use, potentially enhancing overall quality of life and psychophysical well-being. However, it is important to note that while these findings suggest broad applicability, cultural and contextual factors may influence the effectiveness of interventions. Further research is needed to validate the efficacy of these strategies across different populations and settings.

## Data Availability

The original contributions presented in the study are included in the article/supplementary material, further inquiries can be directed to the corresponding authors.

## Footnotes

^1^https://www.wjx.cn/

## References

[ref1] Blake M. J. Trinder J. A. Allen N. B. (2018). Mechanisms underlying the association between insomnia, anxiety, and depression in adolescence: implications for behavioral sleep interventions. Clin. Psychol. Rev. 63, 25–40. doi: 10.1016/j.cpr.2018.05.006, PMID: 29879564

[ref2] Bozoglan B. Demirer V. Sahin I. (2014). Problematic internet use: functions of use, cognitive absorption, and depression. Comput. Hum. Behav. 37, 117–123. doi: 10.1016/j.chb.2014.04.042

[ref3] Brinksma D. M. Dietrich A. Bildt A. D. Buitelaar J. K. Hartman C. A. (2019). ADHD symptoms across adolescence: the role of the family and school climate and the DRD4 and 5-HTTLPR genotype. Eur. Child Adolesc. Psychiatry 29, 1049–1061. doi: 10.1007/s00787-019-01424-3 31628528 PMC7369263

[ref4] Buysse D. J. Iii C. F. R. Monk T. H. Berman S. R. Kupfer D. J. (1989). The Pittsburgh sleep quality index: a new instrument for psychiatric practice and research. Psychiatry Res. 28, 193–213. doi: 10.1016/0165-1781(89)90047-4, PMID: 2748771

[ref5] Cao X. Masood A. Luqman A. Ali A. (2018). Excessive use of mobile social networking sites and poor academic performance: antecedents and consequences from stressor-strain-outcome perspective. Comput. Hum. Behav. 85, 163–174. doi: 10.1016/j.chb.2018.03.023

[ref6] Carvalho-Mendes R. P. Dunster G. P. Iglesia H. O. D. L. Menna-Barreto L. (2020). Afternoon school start times are associated with a lack of both social jetlag and sleep deprivation in adolescents. J. Biol. Rhythm. 35, 377–390. doi: 10.1177/0748730420927603, PMID: 32508224

[ref7] Chen R. (2024). Deeply mediated time and daily life: a sociological study of sleep time. Southeast Acad. Res. 2, 179–190.

[ref8] Chen C. He Z. Xu B. Shao J. Wang D. (2023). A latent profile analysis of sleep disturbance in relation to mental health among college students in China. Front. Public Health 11, 1–9. doi: 10.3389/fpubh.2023.1107692 PMC10266341 37325305

[ref9] Cheng Y. Zhang L. Wang F. Zhang P. Ye B. Liang Y. (2017). The effects of family structure and function on mental health during China’s transition: a cross-sectional analysis. BMC Fam. Pract. 18, 1–8. doi: 10.1186/s12875-017-0630-4 28476107 PMC5420133

[ref10] Chin W. W. Marcolin B. L. Newsted P. R. (2003). A partial least squares latent variable modeling approach for measuring interaction effects: results from a Monte Carlo simulation study and an electronic-mail emotion/adoption study. Inf. Syst. Res. 14, 189–217. doi: 10.1287/isre.14.2.189.16018

[ref11] Chinoy E. D. Duffy J. F. Czeisler C. A. (2018). Unrestricted evening use of light‐emitting tablet computers delays self‐selected bedtime and disrupts circadian timing and alertness. Physiol. Rep. 6:e13692. doi: 10.14814/phy2.13692 29845764 PMC5974725

[ref12] Cohen J. (1988). Statistical power analysis for the behavioral sciences. Mahwah, NJ: Lawrence Erlbaum Associates.

[ref13] Crandall A. Weiss-Laxer N. S. Broadbent E. Holmes E. K. Magnusson B. M. Okano L.. (2020). The family health scale: reliability and validity of a short-and long-form. Front. Public Health 8, 1–13. doi: 10.3389/fpubh.2020.587125 33330329 PMC7717993

[ref15] Davis R. A. (2001). Cognitive-behavioral model of pathological internet use. Comput. Hum. Behav. 17, 187–195. doi: 10.1016/S0747-5632(00)00041-8

[ref16] Delrosso L. M. Mogavero M. P. Ferri R. (2020). Effect of sleep disorders on blood pressure and hypertension in children. Curr. Hypertens. Rep. 22, 1–7. doi: 10.1007/s11906-020-01100-x 32893326

[ref17] Dhir A. Yossatorn Y. Kaur P. Chen S. (2018). Online social media fatigue and psychological wellbeingd psychological wellbeingeinglbeingwellbeinglbeingeingnalysis. Mental health dur. Int. J. Inf. Manag. 40, 141–152. doi: 10.1016/j.ijinfomgt.2018.01.012

[ref18] Gautam P. Dahal M. Baral K. Acharya R. Khanal S. Kasaju A.. (2021). Sleep quality and its correlates among adolescents of western Nepal: a population-based study. Sleep Disorders 2021, 1–8. doi: 10.1155/2021/5590715, PMID: 34055416 PMC8143896

[ref19] Gioia F. Rega V. Boursier V. (2021). Problematic internet use and emotional dysregulation among young people: a literature review. Clin. Neuropsychiatry 18, 41–54. doi: 10.36131/cnfioritieditore20210104, PMID: 34909019 PMC8629046

[ref20] Gong J. Guo S. Hu P. Zhao Y. Tang X. (2024). The relationship between procrastination and sleep quality: the mediating effects of rumination and depression and gender differences. Chin. J. Clin. Psych. 1, 207–212.

[ref21] Haig M. (2018). Notes on a nervous planet. Edinburgh: Canongate Books.

[ref22] Hale L. Emanuele E. James S. (2015). Recent updates in the social and environmental determinants of sleep health. Curr. Sleep Med. Rep. 1, 212–217. doi: 10.1007/s40675-015-0023-y, PMID: 27540510 PMC4987093

[ref23] Hale L. Li X. Hartstein L. E. LeBourgeois M. K. (2019). Media use and sleep in teenagers: what do we know? Curr. Sleep Med. Rep. 5, 128–134. doi: 10.1007/s40675-019-00146-x

[ref24] Hamilton J. L. Chand S. Reinhardt L. Ladouceur C. D. Silk J. S. Moreno M.. (2020). Social media use predicts later sleep timing and greater sleep variability: An ecological momentary assessment study of youth at high and low familial risk for depression. J. Adolesc. 83, 122–130. doi: 10.1016/j.adolescence.2020.07.009, PMID: 32771847 PMC7484414

[ref25] Harbard E. Allen N. B. Trinder J. Bei B. (2016). What's keeping teenagers up? Prebedtime behaviors and actigraphy-assessed sleep over school and vacation. J. Adolesc. Health 58, 426–432. doi: 10.1016/j.jadohealth.2015.12.011, PMID: 26874590

[ref26] Harvey A. G. (2002). A cognitive model of insomniac. Behav. Res. Ther. 40, 869–893. doi: 10.1016/S0005-7967(01)00061-4 12186352

[ref27] Hertenstein E. Feige B. Gmeiner T. Kienzler C. Spiegelhalder K. Johann A.. (2019). Insomnia as a predictor of mental disorders: a systematic review and meta-analysis. Sleep Med. Rev. 43, 96–105. doi: 10.1016/j.smrv.2018.10.006 30537570

[ref28] Higuchi S. Motohashi Y. Liu Y. Maeda A. (2005). Effects of playing a computer game using a bright display on presleep physiological variables, sleep latency, slow wave sleep and REM sleep. J. Sleep Res. 14, 267–273. doi: 10.1111/j.1365-2869.2005.00463.x, PMID: 16120101

[ref29] Huan V. Ang R. Chye S. (2014). Loneliness and shyness in adolescent problematic internet users: the role of social anxiety. Child Youth Care Forum 43, 539–551. doi: 10.1007/s10566-014-9252-3

[ref30] Jahan I. Ismail H. Al Mamun F. Kaggwa M. M. Griffiths M. D. Mamun M. A. (2021). How has the COVID-19 pandemic impacted internet use behaviors and facilitated problematic internet use? A Bangladeshi study. Psychol. Res. Behav. Manag. 14, 1127–1138. doi: 10.2147/PRBM.S323570, PMID: 34345189 PMC8324976

[ref31] Jakimowicz S. Perry L. Lewis J. (2020). Bowen family systems theory: mapping a framework to support critical care nurses' well how how siological var. Nurs. Philos. 22, 1–11. doi: 10.1111/nup.12320 32835447

[ref32] Janet B. Segal L. (1982). Family systems theory: background and implications. J. Commun. 32, 99–107. doi: 10.1111/j.1460-2466.1982.tb02503.x

[ref33] Kansagra S. (2020). Sleep disorders in adolescents. Pediatrics 145, S204–S209. doi: 10.1542/peds.2019-2056I 32358212

[ref35] Koeske G. F. Koeske R. D. (1993). A preliminary test of a stress-strain-outcome model for reconceptualizing the burnout phenomenon. J. Soc. Serv. Res. 17, 107–135. doi: 10.1300/J079v17n03_06

[ref36] Kortesoja L. Vainikainen M. P. Hotulainen R. Merikanto I. (2023). Late-night digital media use in relation to Chronotype, sleep and tiredness on school days in adolescence. J. Youth Adolesc. 52, 419–433. doi: 10.1007/s10964-022-01703-4, PMID: 36401709 PMC9842555

[ref37] Lam L. T. Peng Z. W. (2010). Effect of pathological use of the internet on adolescent mental health: a prospective study. Arch. Pediatr. Adolesc. Med. 164, 1–17. doi: 10.1001/archpediatrics.2010.159 20679157

[ref38] Liang M. Guo L. Huo J. Zhou G. (2021). Prevalence of sleep disturbances in Chinese adolescents: a systematic review and meta-analysis. PLoS One 16, 1–18. doi: 10.1371/journal.pone.0247333 PMC7932116 33661914

[ref39] Lima R. A. de Barros M. V. G. Dos Santos M. A. M. Machado L. Bezerra J. Soares F. C. (2020). The synergic relationship between social anxiety, depressive symptoms, poor sleep quality and body fatness in adolescents. J. Affect. Disord. 260, 200–205. doi: 10.1016/j.jad.2019.08.074, PMID: 31499376

[ref40] Maratia F. Bacaro V. Crocetti E. (2023). Sleep is a family affair: a systematic review and meta-analysis of longitudinal studies on the interplay between adolescentsetsleep and family factors. Int. J. Environ. Res. Public Health 20, 1–25. doi: 10.3390/ijerph20054572 PMC10001512 36901581

[ref41] Mayne S. L. Mitchell J. A. Virudachalam S. Fiks A. G. Williamson A. A. (2021). Neighborhood environments and sleep among children and adolescents: a systematic review. Sleep Med. Rev. 57, 101465–101414. doi: 10.1016/j.smrv.2021.101465 33827031 PMC8164975

[ref42] Mick D. G. Fournier S. (1998). Paradoxes of technology: consumer cognizance, emotions, and coping strategies. J. Consum. Res. 25, 123–143. doi: 10.1086/209531

[ref43] Moretta T. Buodo G. (2020). Problematic internet use and loneliness: how complex is the relationship? A short literature review. Curr. Addict. Rep. 7, 125–136. doi: 10.1007/s40429-020-00305-z

[ref44] Narmandakh A. Roest A. M. de Jonge P. Oldehinkel A. J. (2020). The bidirectional association between sleep problems and anxiety symptoms in adolescents: a TRAILS report. Sleep Med. 67, 39–46. doi: 10.1016/j.sleep.2019.10.018, PMID: 31887607

[ref45] Öks E. Guvenc G. Mumcu S. (2018). Relationship between problematic internet use and time management among nursing students. Comput. Inform. Nurs. 36, 55–61. doi: 10.1097/CIN.0000000000000391 29315092

[ref46] Opakunle T. Aloba O. Opakunle O. Eegunranti B. (2020). Problematic internet use questionnaire-short form-6 (PIUQ-SF-6): dimensionality, validity, reliability, measurement invariance and mean differences across genders and age categories among Nigerian adolescents. Int. J. Ment. Health 49, 229–246. doi: 10.1080/00207411.2020.1776457

[ref47] Otani H. Yoshida S. Morita T. Aoyama M. Kizawa Y. Shima Y.. (2017). Meaningful communication before death, but not present at the time of death itself, is associated with better outcomes on measures of depression and complicated grief among bereaved family members of cancer patients. J. Pain Symptom Manag. 54, 273–279. doi: 10.1016/j.jpainsymman.2017.07.010, PMID: 28711756

[ref48] Pagano M. Bacaro V. Crocetti E. (2023). "Using digital media or sleeping … That is the question". A meta-analysis on digital media use and unhealthy sleep in adolescence. Comput. Hum. Behav. 146, 107813–107816. doi: 10.1016/j.chb.2023.107813

[ref49] Paruthi S. Brooks L. J. D'Ambrosio C. Hall W. A. Kotagal S. Lloyd R. M.. (2016). Recommended amount of sleep for pediatric populations: a consensus statement of the American academy of sleep medicine. J. Clin. Sleep Med. 12, 785–786. doi: 10.5664/jcsm.5866 27250809 PMC4877308

[ref50] Randler C. Wolfgang L. Matt K. Demirhan E. Horzum M. B. Beşoluk Ş. (2016). Smartphone addiction proneness in relation to sleep and morningness–eveningness in German adolescents. J. Behav. Addict. 5, 465–473. doi: 10.1556/2006.5.2016.056 27499228 PMC5264414

[ref51] Ranjan L. K. Gupta P. R. Srivastava M. Gujar N. M. (2021). Problematic internet use and its association with anxiety among undergraduate students. Asian J. Soc. Health Behav. 4, 137–141. doi: 10.4103/shb.shb_30_21

[ref52] Salmela-Aro K. Upadyaya K. Hakkarainen K. Lonka K. Alho K. (2016). The dark side of internet use: two longitudinal studies of excessive internet use, depressive symptoms, school burnout and engagement among Finnish early and late adolescents. J. Youth Adolesc. 46, 343–357. doi: 10.1007/s10964-016-0494-2 27138172

[ref53] Sancho-Domingo C. Carballo J. L. Coloma-Carmona A. Buysse D. J. (2021). Brief version of the Pittsburgh sleep quality index (B-PSQI) and measurement invariance across gender and age in a population-based sample. Psychol. Assess. 33, 111–121. doi: 10.1037/pas0000959, PMID: 33119375

[ref54] Sharman R. Illingworth G. (2019). Adolescent sleep and school performance across gender and age in a popula. Curr. Opin. Physiol. 15, 23–28. doi: 10.1016/j.cophys.2019.11.006

[ref55] Spitzer R. L. Kroenke K. Williams J. B. W. Löwe B. (2006). A brief measure for assessing generalized anxiety disorder: the GAD-7. Arch. Intern. Med. 166, 1092–1097. doi: 10.1001/archinte.166.10.1092 16717171

[ref56] Tandon A. Dhir A. Talwar S. Kaur P. Mntymki M. (2022). Social media induced fear of missing out (FoMO) and phubbing: Behavioural, relational and psychological outcomes. Technol. Forecast. Soc. Chang. 174, 121149–121112. doi: 10.1016/j.techfore.2021.121149

[ref57] Thomas P. A. Liu H. Umberson D. (2017). Family relationships and well-being. Innov. Aging 1, 1–11. doi: 10.1093/geroni/igx025 29795792 PMC5954612

[ref58] Thomée S. Härenstam A. Hagberg M. (2011). Mobile phone use and stress, sleep disturbances, and symptoms of depression among young adults-a prospective cohort study. BMC Public Health 11, 1–11. doi: 10.1186/1471-2458-11-66 21281471 PMC3042390

[ref59] Tochigi M. Usami S. Matamura M. Kitagawa Y. Fukushima M. Yonehara H.. (2016). Annual longitudinal survey at up to five time points reveals reciprocal effects of bedtime delay and depression/anxiety in adolescents. Sleep Med. 17, 81–86. doi: 10.1016/j.sleep.2015.08.024, PMID: 26847979

[ref60] Vernon L. Barber B. L. Modecki K. L. (2015). Adolescent problematic social networking and school experiences: the mediating effects of sleep disruptions and sleep quality. Cyberpsychol. Behav. Soc. Netw. 18, 386–392. doi: 10.1089/cyber.2015.0107, PMID: 26167837

[ref61] Wang Q. Liu Y. J. Wang B. H. An Y. Wang H. L. Zhang Y. Y.. (2022). Problematic internet use and subjective sleep quality among college students in China: results from a pilot study. J. Am. Coll. Heal. 70, 552–560. doi: 10.1080/07448481.2020.1756831, PMID: 32407209

[ref62] Wang D. Sun X. He F. (2023). The mediating effect of family health on the relationship between health literacy and mental health: a national cross sectional survey in China. Int. J. Soc. Psychiatry 69, 1490–1500. doi: 10.1177/00207640231166628, PMID: 37095729

[ref63] Willis T. A. Gregory A. M. (2015). Anxiety disorders and sleep in children and adolescents. Sleep Med. Clin. 10, 125–131. doi: 10.1016/j.jsmc.2015.02.002 26055860

[ref64] Wolniczak I. Caceres-DelAguila J. A. Palma-Ardiles G. Arroyo K. J. SolJ Visscher R. Paredes-Yauri S.. (2013). Association between Facebook dependence and poor sleep quality: a study in a sample of undergraduate students in Peru. PLoS One 8:e59087. doi: 10.1371/journal.pone.0059087 23554978 PMC3595202

[ref65] Xiao H. Shen Y. Zhang W. Lin R. (2023). Applicability of the cognitive model of generalized anxiety disorder to adolescents’ sleep quality: A cross-sectional and longitudinal analysis. Int. J. Clin. Health Psychol. 23:100406. 37663041 10.1016/j.ijchp.2023.100406 PMC10472235

[ref66] Yan Y. Mingyan L. Xiangdong T. Rongmao L. (2010). A review of the relationship between stress response, stress coping, and sleep quality. Adv. Psychol. Sci. 11, 1734–1746. doi: 10.1016/j.ijchp.2023.100406

[ref67] Yeon-Jin K. Hye J. Youngjo L. Donghwan L. Dai-Jin K. (2018). Effects of internet and smartphone addictions on depression and anxiety based on propensity score matching analysis. Int. J. Environ. Res. Public Health 15:859. doi: 10.3390/ijerph15050859 29693641 PMC5981898

[ref68] You Z. Li X. Ye N. Zhang L. (2021). Understanding the effect of rumination on sleep quality: a mediation model of negative affect and bedtime procrastination. Curr. Psychol. 8, 136–144. doi: 10.1007/s12144-020-01337-4

[ref69] Zhang L. G. Cheng L. F. Wang T. T. Wang L. L. Zhou S. J. Luo Y. H.. (2023). Chain mediating effect of insomnia, depression, and anxiety on the relationship between nightmares and cognitive deficits in adolescents. J. Affect. Disord. 322, 2–8. doi: 10.1016/j.jad.2022.10.047, PMID: 36343783

[ref70] Zhang Y. Huang Z. Zhang M. Li C. Zhao Z. Zhang X.. (2023). Sleep status among children and adolescents aged 6-17 years-China, 2016-2017. China CDC Weekly 5, 11–16. doi: 10.46234/ccdcw2023.003 36777469 PMC9902748

[ref71] Zhao X. Lynch J. G. Chen Q. (2010). Reconsidering baron and Kenny: myths and truths about mediation analysis. J. Consum. Res. 37, 197–206. doi: 10.1086/651257

